# Proteomics of Vitreous Humor of Patients with Exudative Age-Related Macular Degeneration

**DOI:** 10.1371/journal.pone.0096895

**Published:** 2014-05-14

**Authors:** Michael Janusz Koss, Janosch Hoffmann, Nauke Nguyen, Marcel Pfister, Harald Mischak, William Mullen, Holger Husi, Robert Rejdak, Frank Koch, Joachim Jankowski, Katharina Krueger, Thomas Bertelmann, Julie Klein, Joost P. Schanstra, Justyna Siwy

**Affiliations:** 1 Department of Ophthalmology, Goethe University, Frankfurt am Main, Germany; 2 Doheny Eye Institute, Los Angeles, California, United States of America; 3 Department of Ophthalmology, Ruprecht Karls University, Heidelberg, Germany; 4 Mosaiques Diagnostics, Hannover, Germany; 5 BHF Glasgow Cardiovascular Research Centre, University of Glasgow, Glasgow, United Kingdom; 6 Department of General Ophthalmology, Lublin University, Poland; 7 Department of Nephrology, Endocrinology, and Transplantation Medicine Charité-Universitaetsmedizin, Berlin, Germany; 8 Department of Ophthalmology, Philipps University, Marburg, Germany; 9 Institut National de la Santé et de la Recherche Médicale (INSERM), U1048, Institut of Cardiovascular and Metabolic Disease, Toulouse, France; 10 Université Toulouse III Paul-Sabatier, Toulouse, France; Bascom Palmer Eye Institute, University of Miami School of Medicine, United States of America

## Abstract

**Background:**

There is absence of specific biomarkers and an incomplete understanding of the pathophysiology of exudative age-related macular degeneration (AMD).

**Methods and Findings:**

Eighty-eight vitreous samples (73 from patients with treatment naïve AMD and 15 control samples from patients with idiopathic floaters) were analyzed with capillary electrophoresis coupled to mass spectrometry in this retrospective case series to define potential candidate protein markers of AMD. Nineteen proteins were found to be upregulated in vitreous of AMD patients. Most of the proteins were plasma derived and involved in biological (ion) transport, acute phase inflammatory reaction, and blood coagulation. A number of proteins have not been previously associated to AMD including alpha-1-antitrypsin, fibrinogen alpha chain and prostaglandin H2-D isomerase. Alpha-1-antitrypsin was validated in vitreous of an independent set of AMD patients using Western blot analysis. Further systems biology analysis of the data indicated that the observed proteomic changes may reflect upregulation of immune response and complement activity.

**Conclusions:**

Proteome analysis of vitreous samples from patients with AMD, which underwent an intravitreal combination therapy including a core vitrectomy, steroids and bevacizumab, revealed apparent AMD-specific proteomic changes. The identified AMD-associated proteins provide some insight into the pathophysiological changes associated with AMD.

## Introduction

Age dependent alterations of the retinal pigment epithelium (RPE) and its basal membrane, called Bruchs membrane, are widely accepted as the main pathophysiological reason for age-related macular degeneration (AMD) and is thus the leading cause of blindness in people over the age of 60 years in industrialized countries [1]. The upregulation of vascular endothelial growth factor (VEGF) and the development of a choroidal neovascularization (CNV) are the blueprint for the conversion to the exudative or wet AMD form. Our understanding today of the disease and the interaction of intravitreal anti-VEGF treatment is thereby coined and determined by clinical diagnostics, mainly optical coherence tomography and fluorescein angiography. Since AMD is a pure retinochoroidal disease, circulating *in vivo* biomarkers such as HbA1c in the diagnosis and treatment of diabetes are still absent for AMD. Samples from the human vitreous might best qualify as a source of biomarkers for AMD due to the proximity to the retina and the efflux of cytokines into the vitreous cavity [2]. However, most published protein analyses in exudative AMD derive from experimental animal models, *ex vivo* samples or *in vivo* from ocular anterior chamber aspirates (AC) with the incorporated flaws [3], [4], [5], [6]. Ecker et al. demonstrated, that cytokine and growth factor levels from the AC do not reliably reflect those levels found in the vitreous and thus it is questionable to assess the activity of a purely retinochoroidal disease by examining an AC aspirate [2], [7], [8]. But results from vitreous samples in AMD are scarce and published data differs on patient selection, sampling technique and analysis method [7], [9], [10], [11], [12], [13], [14], [15].

Proteome analysis allows the simultaneous assessment of a large number of proteins in a sample. Proteome analyses have been performed in a variety of ocular diseases, including primary open-angle glaucoma and cataract [16], [17], [18]. Further exploration of vitreous protein profiles was performed even tough clinical factors, like consistency of the vitreous, length of the eye, attachment of the posterior hyaloid are to date neglected in the literature [19]. Especially in the context of wet AMD the current proteomic data on vitreous or aqueous humor are incomplete.

Capillary electrophoresis coupled to mass spectrometry (CE-MS) is a powerful and very reproducible technology platform with known performance characteristics [20]. This automated, sensitive, fast proteome analysis technique [21] using CE as a front-end fractionation coupled to mass spectrometry, separates peptides and small proteins (<20 kDa) based on migration in the electrical field with high resolution in a single step. It enables analysis of thousands of peptides per sample using a sub-microliter sample volume and it has been used in numerous clinical biomarker studies, mostly examining urine as the specimen of interest [22], [23].

In this pilot study, we performed a bottom-up analysis combing the reproducibility of CE-MS for selection of candidate marker proteins, and LC-MS/MS for sequence identification of these markers in vitreous of 73 AMD patients and controls. This led to the identification of a number of candidate proteins not previously shown to be involved in AMD. Systems biology analysis of the data suggested an increase in immune response, complement activation and protease activity to be involved in the pathophysiology of AMD.

## Material and Methods

### Sampling and patient characteristics

Vitreous samples were acquired at the beginning of an intravitreal combination treatment for wet AMD, which involved a 23-gauge core vitrectomy of at least 4 cc vitreous, before the application of bevacizumab, triamcinolone, and dexamethasone [22]. The same surgical technique was applied for the removal of idiopathic vitreous floaters, substituting balanced salt solution (BSS Plus, Alcon, Freiburg, Germany). This study adhered to the Declaration of Helsinki and was approved by the Investigational Review Board of the Goethe University. Written informed consent was obtained from all participants, explaining the risks and benefits of the treatment (the advantage of a combination treatment for wet AMD rather than with anti-VEGF monotherapy is summarized here [24], [25], [26]; the vitreous samples would otherwise have been disposed).

A total of 88 undiluted and previously untreated (any intraviteal drug application) samples were analyzed (**Table 1**). The 73 AMD samples came from 73 patients (50 women 23 men) with a mean age 77.8 ± 8.9 years (standard deviation). Sixteen of the 73 patients had a hemorrhagic CNV; 37 had an active CNV (10 with signs accompanying bleeding); 13 had a CNV and greater than 80% fibrous staining in the fluorescein angiography (FA); and 7 had a CNV-associated RPE detachment and no intraretinal fluid. These classifications were assigned after a complete ocular examination, which included a slit-lamp biomicroscopy, indirect ophthalmoscopy, color fundus photography, spectral domain optical coherence tomography (3D-OCT 2000, Topcon, Willich, Germany), and FA. Patients with previous intravitreal anti-VEGF treatment, including intraocular steroids, or systemic diabetes, nephropathy or uncontrolled hypertension were excluded. Patients with any other compromising ocular condition, such as diabetic retinopathy or uveitis, were also considered ineligible for this study.

**Table 1:** Epidemiology of the samples.

			Number	Age (± SD)	Sex		Eye	
					F	M	RE	LE
AMD	Hemorrhagic CNV Bleeding		16	80.8 ± 9.0	15	1	6	10
	CNV	With blood signs	10	77.7 ± 10.5	4	6	8	2
		Without blood signs	27	77.2 ± 7.4	17	10	12	15
	Fibrous		13	75.4 ± 8.1	9	4	8	5
	RPE-Detachment		7	78.3 ± 12.9	5	2	4	3
Control			15	60.0 ± 16.0	8	7	6	9

SD  =  standard deviation, F  =  female, M  =  male, RE/LE  =  right/left eye, CNV  =  choroidal neovascularization, AMD  =  age related macular degeneration, RPE  =  retinal pigment epithelium.

All patients were recruited from the retina clinic of the department of ophthalmology from the Goethe University in Frankfurt am Main in Germany. Vitreous samples from 15 patients with idiopathic floaters (8 women, 7 men; mean age 60 ± 16 years) served as controls. All vitreous samples were stored at −80°C.

### Tryptic digestion of vitreous

A 10-µL portion of the thawed sample was diluted with 90 µL 0.1% SDS, 20 mM DTT, and 0.1 M TrisHCl (pH = 7.6). The sample was sonicated at room temperature for 30 minutes to decrease viscosity and break up hyaluronic polymers contained in the vitreous humor. This was followed by denaturation at 95°C for 3 min. Samples were subsequently incubated with 80 mM Iodoacetamide at room temperature for 30 min in the absence of light, followed by the addition of ammonium bicarbonate buffer solution (300 µL, 50 mM) and applied to NAP-5 columns equilibrated in 50 mM ammonium bicarbonate buffer solution.

Twenty µg of Lyophilized trypsin was dissolved in 50 µL of buffer solution provided with the lypholized product. Two µL of this solution was added to the desalted sample. Trypsin digestion was carried out overnight at a temperature of 37°C. Subsequently the samples were lyophilized, stored at 4°C and resuspendend in HPLC-grade H_2_O shortly before mass spectrometry analysis.

### CE-MS analysis

CE-MS analysis was performed as described by Theodorescu et al [27]. A P/ACE MDQ capillary electrophoresis system (Beckman Coulter, Brea, CA) was linked online to a micro-TOF MS (Bruker Daltonik, Leipzig, Germany). The sprayer (Agilent Technologies, Santa Clara, CA) interfacing the CE and MS was grounded and the interface potential was adjusted to −4.5 kV. Signals were recorded at an m/z range of 350–3000. The detection limit of the TOF-Analyzer is in the range of 1 fmol [27].

### Data processing

Analysis of raw CE-MS data was carried out using MosaiquesVisu [28]. MosaiquesVisu uses isotope identification and conjugated mass detection for mass deconvolution. Signals with a signal-to-random noise ratio >4 and charge >1 were used. Mass spectral ion peaks from the same molecule at different charge states were deconvoluted into a single mass.

In total, 292 signals for mass and CE-time with a frequency ≥35% could be determined that served as reference signals for normalization of peptide CE-time and mass using linear regression. For signal intensity normalization, 22 internal standards were selected that were consistently detected in vitreous samples (average frequency 83%) and that did not appear to be significantly associated with the disease. This signal intensity normalization using internal standards has been shown to be a reliable method to address both analytical and biological variances in biological samples [29].

The normalized peptides were deposited, matched, and annotated in a Microsoft SQL database. Peptides were considered identical when deviation of mass was < ±50 ppm for an 800 Da peptide. The mass deviation was adjusted by an increase in size of up to ±75 ppm for 15 kDa. Peptides were considered identical if the CE-migration time window did not exceed 2–5%, continuously increasing between 19 and 50 min. A number of peptides were only sporadically observed. To eliminate such low relevance peptides, all peptides that appeared only once were removed and were not considered for further analysis.

### Tandem mass spectrometry (MS/MS) sequencing

For MS/MS analysis five lyophilized, tryptic-digested randomly selected vitreous samples were dissolved in 15 µL distilled water. Fractionation was carried out according to Metzger et al. [30] using a Dionex Ultimate 3000 Nano LC System (Dionex, Camberly, UK). After loading (5 µl) onto a Dionex 0.1×20 mm 5 µm C18 nano trap column at a flowrate of 5 µl/min in 98% 0.1% formic acid and 2% acetonitrile, sample was eluted onto an Acclaim PepMap C18 nano column 75 µm×15 cm, 2 µm 100 Å at a flow rate of 0.3 µl/min. The trap and nano flow column were maintained at 35°C. The samples were eluted with a gradient of solvent A:98% 0.1% formic acid, 2% acetonitrile verses solvent B: 80% acetonitrile, 20% 0.1% formic acid starting at 1% B for 5 minutes rising to 20% B after 90 min and finally to 40%B after 120 min. The column was then washed and re-equilibrated prior to the next injection. The eluant was ionized using a Proxeon nano spray ESI source operating in positive ion mode into an Orbitrap Velos FTMS (Thermo Finnigan, Bremen, Germany). Ionization voltage was 2.6 kV and the capillary temperature was 200°C. The mass spectrometer was operated in MS/MS mode scanning from 380 to 2000 amu. The top 20 multiply charged ions were selected from each scan for MS/MS analysis using HCD at 40% collision energy. The resolution of ions in MS1 was 60,000 and 7,500 for HCD MS2. The MS data and the human, non redundant database IPI were matched using the SEQUEST software. Trypsin was used as the enzyme while screening for proteins. Hydroxylated proline from collagen fragments and oxidation of methionine were accepted as variable modifications and carbamidomethylated cystein as fixed modification. A maximal mass deviation of 10 ppm for MS and 0.8 Da for MS/MS was accepted. Only proteins represented by a minimum two peptides were accepted in LC-MS/MS analysis. The sequences were matched to the detected CE-MS data as described by Zürbig et al [19]. Although in this matching procedure the LC retention time can not be used, CE-MS data generate an additional parameter that can be used in this matching procedure, which is the charge of the peptides (at low pH, the condition in which peptides are analysed by CE-MS). Therefore, even when having identical or very close masses in the LC-MS/MS analysis, discrimination between peptides with similar masses can be performed on the basis of their charge. This is due to the fact that the number of basic and neutral polar amino acids of peptide sequences distinctly correlates with their CE-MS migration time/molecular weight coordinates. In nearly all cases this allows linking a unique LC-MS/MS peptide to a CE-MS peptide as shown previously [19].

CE-MS peptides with sequencing information were combined for each protein. Protein abundance was calculated as the average of all normalized CE-MS peptide intensities for the given protein. Mean protein abundance in the case group was compared to the mean protein abundance in the control group. Protein entries were mapped to the SwissProt database using either the mapping service provided by UniProt or via Blast searching (web.expasy.org/blast/) and merged according to the SwissProt names.

### Biomarker definition

Candidate AMD biomarkers were defined by examination of differences in frequency and signal intensity of the proteins between the AMD patients and controls. Mean CE-MS based protein signal intensity was used as a measure for relative abundance. Statistical analysis was performed using Graph Pad Prism 5.0 software. A F-test was performed to test for data distribution. When data were normally distributed, a parametric t-test was performed; otherwise, the statistical analysis was performed using a Mann-Whitney test or Wilcoxon signed rank test. Multiple hypotheses testing correction was performed using the Benjamini-Hochberg test for false discovery rate [31].

### Bioinformatics analysis

Gene ontology (GO) keyword-cluster analysis was done using CytoScape (www.cytoscape.org) and the ClueGO plug-in, where statistically significant molecules with p-values of less than 0.05 were compared. The interactome analysis was performed using CytoScape and the Michigan Molecular Interactor plug-in (mimi.ncibi.org). Full interactome analysis was carried out by connecting molecules through neighboring proteins, whereas the condensed protein-protein interaction analysis was performed by searching for direct associations. The paradigm of this analysis is that molecules with similar cellular involvement tend to cluster together through physical interactions, such as molecular machines. The disease analysis is based on known or inferred genetic disorders associated with mutations of specific genes using the Online Mendelian Inheritance in Man database for data mining. An additional pathway analysis was carried out using the Kyoto encyclopedia of genes and genomes (KEGG) database, where KEGG accession numbers of the statistically significant molecules were used for mapping-queries. Additionally, disease-specific descriptions were retrieved from the Online Mendelian Inheritance in Man (OMIM) database as well as the UniProt database. Cellular expression data was also obtained from the latter resource.

### Western blot analysis

To determine the protein levels of Transthyretin, Apolipoprotein A 1, Alpha-1 Antitrypsin, Serotransferrin and Retinol-binding protein 3 we obtained vitreous fluid as described above. The extracted proteins (20 µl/5 µg protein) were loaded and subjected to 12.5% sodium dodecyl sulfate-polyacrylamide gel electrophoresis (SDS-PAGE) for 30 min at 100 V and then 90 min at 150 V and subsequently transferred onto nitrocellulose membranes using Trans-Blot Turbo Transfer System (Bio-Rad, Hercules, CA, USA). The membranes were blocked for 1 h at room temperature in blocking buffer consisting of 5% BSA in PBS and 0.1% Tween. This was followed by a 4×5 min washing procedure in Wash Buffer (PBS + 0.1% Tween). Blots were incubated with primary antibodies (Santa Cruz Biotechnology) for 2 h at 4°C followed by incubation with secondary antibodies conjugated to horseradish peroxidase (Santa Cruz Biotechnology) for 2 h at 4°C. Blots were visualized with enhanced chemiluminescence (Amersham Biosciences, Piscataway, NJ, USA).

## Results

### Sequencing of tryptic peptides

Using LC-MS/MS, 622 of the tryptic peptides detected with CE-MS could be identified. The mass of sequenced peptides ranged between 804 and 3953 Da. These tryptic peptides corresponded to 97 different proteins in vitreous humor (**Table 2**).

**Table 2:** Proteins in vitreous humor detected by CE-MS and identified by LC-MS/MS analysis.

Protein	UniProt*	Peptide number**	Coverage*** (%)	Peptide number control****	Peptide number case****
Actin, aortic smooth muscle	P62736	1	3	0	1
Afamin	P43652	2	4	2	2
Angiotensinogen	P01019	1	2	1	1
Alpha-1-acid glycoprotein 1	P02763	7	27	7	7
Alpha-1-acid glycoprotein 2	P19652	3	15	3	3
Alpha-1-antitrypsin	P01009	20	52	20	20
Alpha-1B-glycoprotein	P04217	5	11	5	5
Alpha-2-HS-glycoprotein	P02765	4	16	4	4
Alpha-2-macroglobulin	P01023	13	10	9	13
Alpha-crystallin B chain	P02511	2	14	1	2
Amyloid-like protein 2	Q06481	2	4	2	2
Antithrombin-III	P01008	10	23	4	10
Apolipoprotein E	P02649	13	43	12	13
Apolipoprotein A-I	P02647	18	63	16	18
Apolipoprotein A-II	P02652	4	41	4	4
Apolipoprotein A-IV	P06727	10	30	5	10
Beta-2-microglobulin	P61769	2	17	1	2
Beta-crystallin B2	P43320	11	50	11	11
Chitinase-3-like protein 1	P36222	1	3	1	1
Ceruloplasmin	P00450	14	20	12	14
Clusterin	P10909	12	31	11	12
Collagen alpha-1(I) chain	P02452	2	1	2	2
Collagen alpha-1(II) chain	P02458	38	27	35	36
Collagen alpha-1(III) chain	P02461	1	2	1	1
Collagen alpha-1(IX) chain	P20849	5	9	4	5
Collagen alpha-1(V) chain	P20908	4	1	2	3
Collagen alpha-1(XI) chain	P12107	5	3	5	5
Collagen alpha-1(XII) chain	Q99715	1	1	1	1
Collagen alpha-1(XXII) chain	Q8NFW1	1	1	1	1
Collagen alpha-1(XXIII) chain	Q86Y22	1	3	1	1
Collagen alpha-1(XXVIII) chain	Q2UY09	1	2	0	1
Collagen alpha-2(IX) chain	Q14055	4	6	4	4
Collagen alpha-2(XI) chain	P13942	1	2	1	1
Collagen alpha-3(IX) chain	Q14050	5	7	5	5
Complement C3	P01024	32	23	23	32
Complement C4-B	P0C0L5	11	7	10	11
Complement factor B	P00751	5	7	3	5
Alpha-crystallin A chain	P02489	3	19	3	3
Cathepsin D	P07339	3	11	3	3
Cystatin-C	P01034	2	18	2	2
Dermcidin	P81605	3	23	1	3
Dickkopf-related protein 3	Q9UBP4	6	22	6	6
Double-strand break repair protein MRE11A	P49959	1	2	0	1
Fibrinogen alpha chain	P02671	4	6	3	4
Fibrinogen beta chain	P02675	1	3	1	1
Gelsolin	P06396	1	2	0	1
Glutathione peroxidase 3	P22352	4	23	3	4
Haptoglobin	P00738	13	24	10	13
Hemoglobin subunit beta	P68871	1	9	1	1
Hemopexin	P02790	14	29	12	14
Heparin cofactor 2	P05546	1	2	1	1
Histidine-rich glycoprotein	P04196	2	4	2	2
Ig alpha-1 chain C region	P01876	3	8	3	3
Ig alpha-2 chain C region	P01877	4	8	2	2
Ig gamma-1 chain C region	P01857	11	37	11	11
Ig gamma-3 chain C region	P01860	4	12	3	4
Ig heavy chain V-III region GAL	P01781	2	8	1	2
Ig heavy chain V-III region TRO	P01762	1	6	1	1
Ig kappa chain V-I region EU	P01598	1	17	1	1
Ig kappa chain V-III region SIE	P01620	1	17	1	1
Ig kappa chain C region	P01834	5	80	4	5
Ig lambda-2 chain C regions	P0CG05	3	42	3	3
IgGFc-binding protein	Q9Y6R7	4	1	4	4
Immunoglobulin lambda-like polypeptide 5	B9A064	1	9	1	1
Inter-alpha-trypsin inhibitor heavy chain H1	P19827	3	4	3	3
Inter-alpha-trypsin inhibitor heavy chain H4	Q14624	2	2	1	2
Keratin, type I cytoskeletal 10	P13645	18	34	16	18
Keratin, type I cytoskeletal 14	P02533	3	7	2	3
Keratin, type I cytoskeletal 9	P35527	7	14	6	7
Keratin, type II cytoskeletal 1	P04264	18	26	17	17
Keratin, type II cytoskeletal 2 epidermal	P35908	3	6	3	3
Keratin, type II cytoskeletal 5	P13647	1	2	1	1
Keratin, type II cytoskeletal 6A	P02538	1	2	1	1
Keratin, type II cytoskeletal 6B	P04259	2	4	2	2
Kininogen-1	P01042	4	5	2	4
Leucine-rich alpha-2-glycoprotein	P02750	1	3	0	1
Opticin	Q9UBM4	3	9	2	3
Osteopontin	P10451	7	34	7	7
Pigment epithelium-derived factor	P36955	12	31	11	12
Plasminogen	P00747	1	1	0	1
Prostaglandin-H2 D-isomerase	P41222	4	21	4	4
Protein Jade-2	Q9NQC1	1	1	1	1
Protein S100-A7	P31151	1	11	1	1
Protein S100-A9	P06702	2	18	2	2
Prothrombin	P00734	2	4	2	2
Retinol-binding protein 3	P10745	15	18	14	15
Ig kappa chain V-III region VG	P04433	2	23	1	2
Plasma protease C1 inhibitor	P05155	5	12	5	5
Serotransferrin	P02787	44	55	43	44
Alpha-1-antichymotrypsin	P01011	15	36	12	15
Serum albumin	P02768	55	75	51	55
Complement C4-A	P0C0L4	1	1	1	1
Titin	Q8WZ42	1	0	0	1
Transthyretin	P02766	7	63	7	7
Vitamin D-binding protein	P02774	5	9	4	5
Vitronectin	P04004	3	9	2	3
Zinc-alpha-2-glycoprotein	P25311	2	9	2	2

*Uniprot accession numbers that can be found on www.uniprot.org; ** Number of peptides observed by CE-MS analysis and sequenced by LC-MS/MS for each identified protein; *** Percentage of peptide coverage of the protein sequence; ****, Number of peptides observed by CE-MS and sequenced by LC-MS/MS in controls or cases.

### Definition of AMD-specific proteins

**Figure 1:**
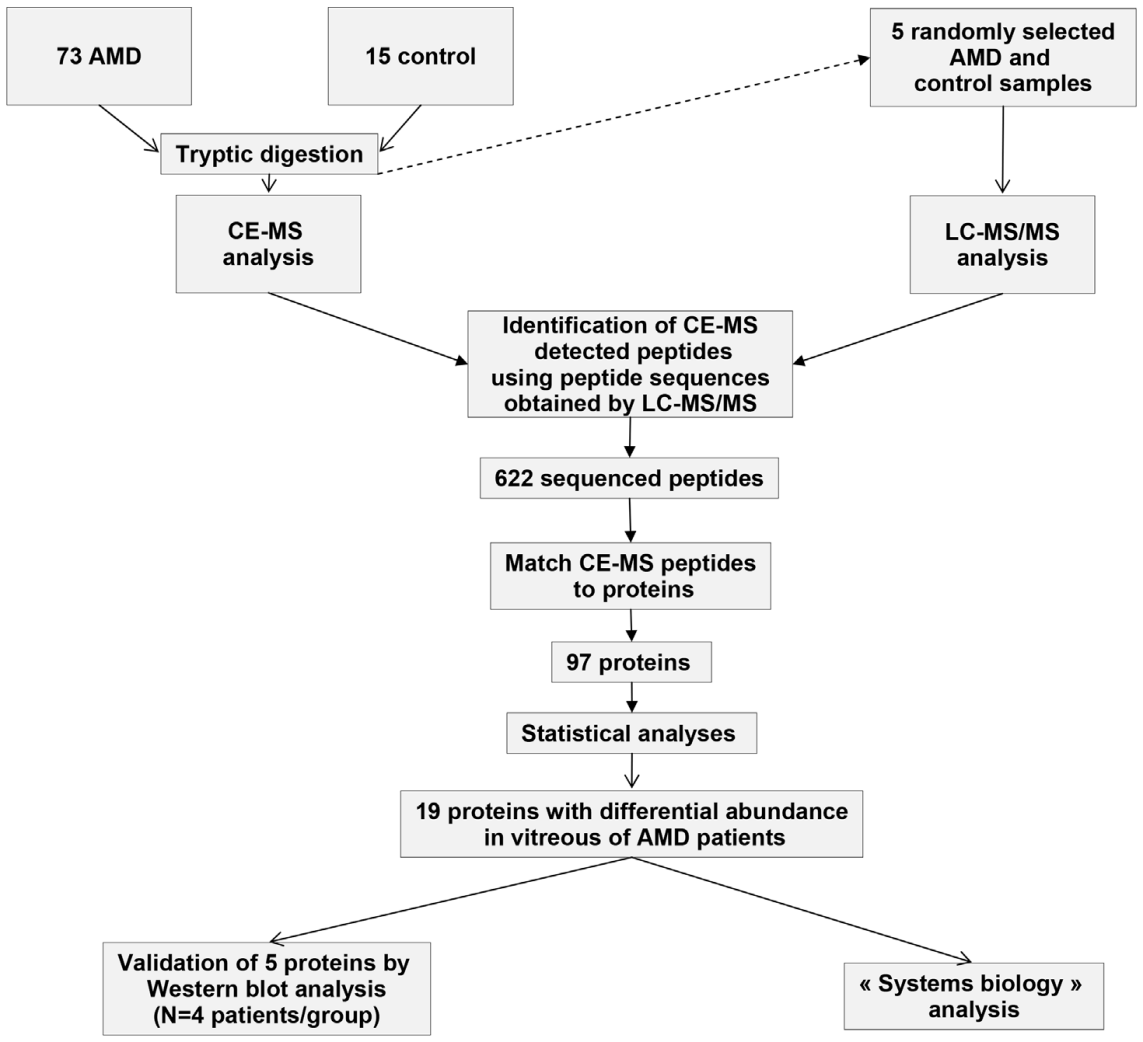
Study design and results.

**Figure 1** is a descriptive presentation of the study setup and findings. All samples were analysed by CE-MS. Next, for statistical analysis, CE-MS detected tryptic peptides were matched to the sequences identified by LC-MS/MS. 622 of the CE-MS detected peptides could be identified by their amino acid sequence. These peptides were combined to 97 proteins as described [29] and this protein distribution was statistically analysed. 19 proteins displayed significant differential abundance in vitreous (**Table 3**). All proteins with a p-value of <0.05 were found to be upregulated in the AMD population.

**Table 3:** List of significant regulated proteins.

Protein name	Fold change AMD/control	Standard deviation for fold change	p-value	adjusted p-value
Ig kappa/lambda chain C region	6.56	13.27	**4.58E-06**	**4.45E-04**
Serum albumin	1.91	1.49	**3.27E-05**	**1.58E-03**
Ig gamma-1 chain C region	3.14	4.70	**6.54E-05**	**1.74E-03**
Antithrombin-III	5.50	12.29	**7.16E-05**	**1.74E-03**
Ig lambda-2 chain C regions	4.55	9.94	**1.30E-04**	**2.51E-03**
Serotransferrin*	1.74	1.52	**3.99E-04**	**6.45E-03**
Afamin	3.28	6.58	**1.93E-03**	**2.16E-02**
Histidine-rich glycoprotein	10.85	49.18	**2.45E-03**	**2.16E-02**
Retinol-binding protein 3*	2.78	5.51	**2.72E-03**	**2.20E-02**
Apolipoprotein A-I*	2.30	3.62	**3.88E-03**	**2.73E-02**
Fibrinogen alpha chain	2.90	6.30	**3.94E-03**	**2.73E-02**
Ig alpha-1 chain C region	35.34	147.90	**2.32E-03**	**2.16E-02**
Alpha-2-HS-glycoprotein	2.58	5.78	**1.55E-02**	8.70E-02
Transthyretin*	1.74	2.11	**1.61E-02**	8.70E-02
Prostaglandin-H2 D-isomerase	1.65	1.97	**2.33E-02**	1.19E-01
Haptoglobin	2.92	7.89	**3.34E-02**	1.46E-01
Glutathione peroxidase 3	4.26	18.42	**3.79E-02**	1.51E-01
Alpha-1-antitrypsin*	1.73	2.56	**4.04E-02**	1.51E-01
Inter-alpha-trypsin inhibitor heavy chain H1	2.34	5.84	**4.82E-02**	1.67E-01

Legend: Fold change AMD/control. Fold increase or decrease observed in AMD patients compared to controls; p-value - unadjusted p-value (Wilcoxon signed-rank test); adjusted p-value - p-value corrected for multiple testing (Benjamini and Hochberg method) and expressed in bold, when statistically significant. * proteins selected for western-blot analysis.

### Validation using Western blot analysis

**Figure 2:**
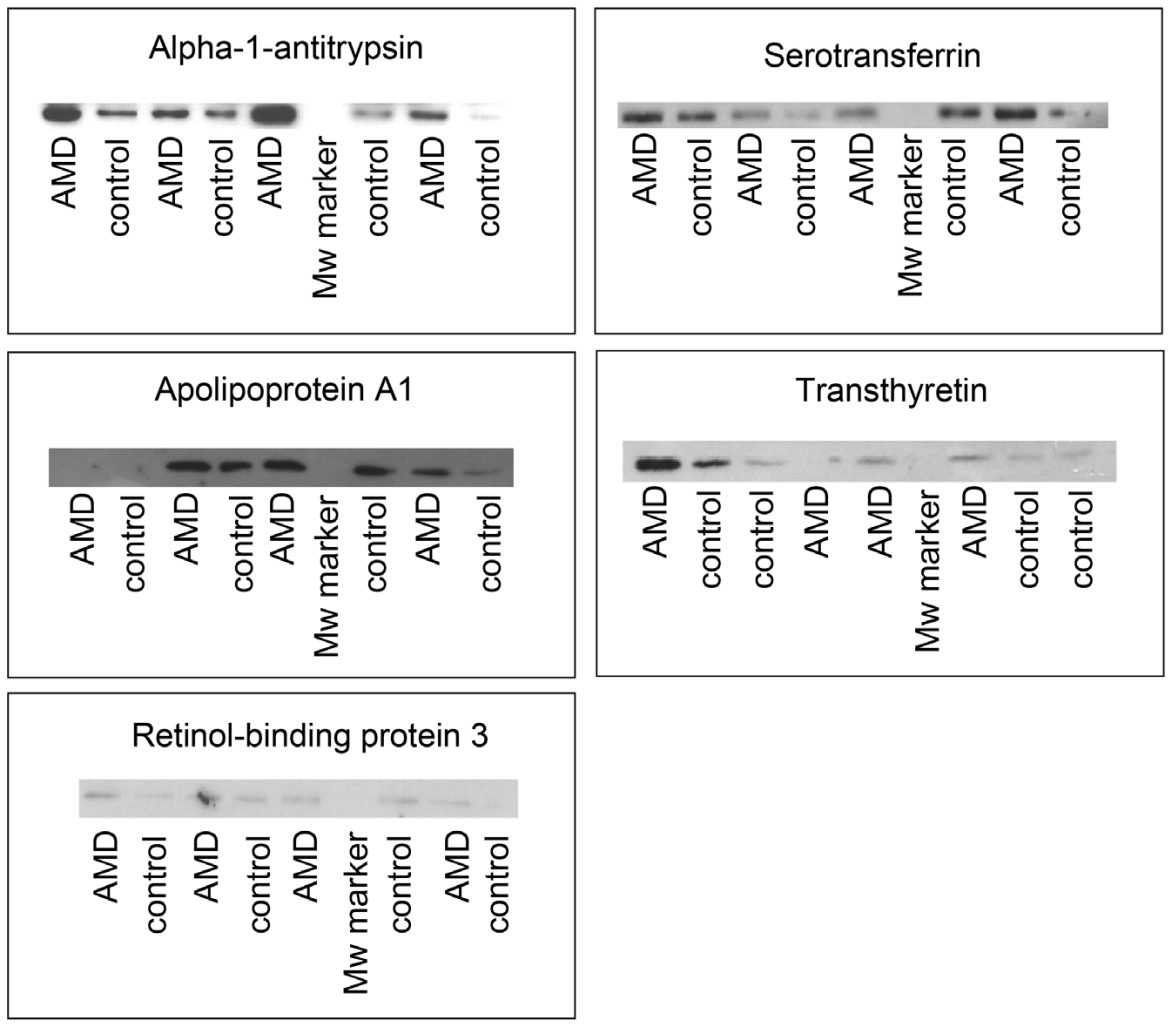
Western blot validation of candidate markers for AMD. Representative Western blots of analysis of expression of selected vitreous proteins.

Western blot analysis was used to verify the findings in **table 3** on 5 randomly selected proteins for which antibodies were readily available, using a new but small set of AMD and control samples (n = 4/group). Four out of the 5 proteins analyzed did not display a significant variation, but the fold increases for apolipoprotein A1 and transthyretin suggested increased expression in vitreous of AMD patients similarly to what observed in the intial CE-MS experiments. The absence of significance is most probably due to the small patient population used for in the validation experiments (**Table 4** and **Figure 2**). However, we validated increased alpha-1-antitrypsin abundance in vitreous of AMD patients (p = 0.02).

**Table 4:** Western blot analysis of selected proteins found upregulated in vitreous of AMD patients by CE-MS.

Protein name	AMD	control	P-value	Fold change	Regulation in proteome analysis
Alpha-1-Antitrypsin	1618.6±610.2	689.5±174.1	p = 0.02	2.35	↑
Apolipoprotein A 1	1925.3±404.9	1463.2±360.4	p = 0.27	1.32	↑
Retinol-binding protein 3	471.9±50.9	427.3±53.9	p = 0.28	1.10	↑
Serotransferrin	1224.1±231.1	1059.6±247.7	p = 0.25	1.16	↑
Transthyretin	1169.8±592.8	747.4±143.8	p = 0.15	1.57	↑

Presented are the results of intensities measured by enhanced chemiluminescence from SDS PAGE Western blots. The mean ± standard deviation from 4 independent undiluted vitreous AMD and 4 independent undiluted vitreous control samples is given. P-values were calculated by the Mann-Whitney statistical test.

### Gene enrichment and pathway analysis

**Figure 3:**
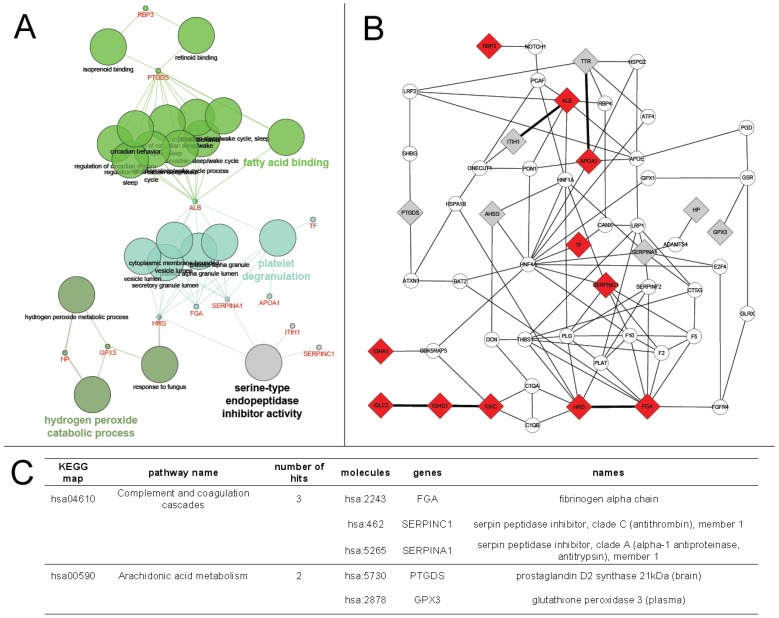
Bioinformatic analysis of identified biomarkers. **A.** Gene ontology analysis shows proteins involved in fatty acid binding. platelet degranulation. serine protease inhibitor activity and hydrogen peroxide catabolic processes. **B** Interaction network of identified biomarker candidates involving 18 out of the 19 proteins. Proteins involved in inflammation. acute phase response (including cellular adhesion). signaling via lipid-mediated pathways (including transport) and activation of proteolytic cascades. as well as transcriptional activity are indicated. Red diamonds indicate proteins which are significant after correction for multiple testing. and grey ones are the remainder of the query set. Circles indicate gap-fillers which were added to connect proteins via protein-protein interactions. Direct association between the significant biomarker set are indicated by a bold line. and relate to immune response (immunoglobulin cluster). protease inhibitor activity. and an activation of the peroxisome proliferator-activated receptor signaling pathway/CDC42 signal transduction pathway. as suggested through APOA1 interactions. **C.** Kyoto encyclopedia of genes and genomes pathway analysis. Statistically relevant biomarker proteins were mapped onto KEGG pathway maps and showed an involvement of fibril formation and inhibition of fibrinolysis in the coagulation cascade and association with arachidonic acid metabolism.

The 19 proteins with differential abundance in AMD were analyzed for their biological process, molecular function and the cellular component. GO cluster analysis using ClueGO showed that these proteins are primarily involved in biological (ion) transport and secretion/exocytosis (platelet degranulation (ALB, TF, APOA1, SERPINA1, HRG, FGA) and fatty acid binding (RBP3, ALB, PTGDS)), protease inhibitor activity (ITIH1, SERPINA1, SERPINC1, HRG), and processes involving hydrogen peroxide (GPX3, HP). These molecules consist mostly of secreted proteins (**Figure 3A**).

A global interactome analysis of the statistically relevant proteins using the Michigan Molecular Interactor plug-in, which mines data from protein-protein, protein-gene, gene-gene, molecule-pathway, and molecule-keyword associations, resulted in a densly interconnected network (**Figure 3B**). This expanded network of 54 proteins, including molecules known to be associated with the query molecules, consists of an immunoglobulin-cluster and complement activation (containing IGHA1, IGKC, IGHG1, IGLC2, AHSG, ITIH1, and FGA), suggesting inflammation and acute phase response processes being activated in the original source tissue, and processes including cell adhesion, lipid metabolism (RBP3, PTGDS), transport (APOA1, ALB, TTR, TF), anti-apoptosis (GPX3), and proteolysis (proteases (HP) and protease inhibitors (HRG, SERPINA1 and SERPINC1)). Inter-linking molecules suggest an involvement of transcriptional elements such as ONECUT and HNF1A/4A, which are modulators of genes involved in lipid metabolic processes, blood coagulation

Analysis of the condensed network of direct interactions between the 19 proteins shows the immunoglobulin cluster containing IGLC2, IGKC, and IGHG1, binary interactions between ALB and ITIH1, and between HRG and FGA, as well as a binary cluster of APOA1 and TTR. The latter suggests that the peroxisome proliferator-activated receptor (PPAR) signaling pathway is perturbed in AMD through an association with TTR and fibrils **(** **Figure 3B**).

Data mining of the Online Mendelian Inheritance in Man database by associated disease clustering showed one relevant cluster consisting of APOA1 (cataract formation) and TTR (fibril formation (neurodegenerative)). The same components were also found by association using a similar approach to mine the disease entries in the KEGG database, where the common denominator was found as familial amyloidosis (KEGG disease entry H00845), linking it to the complement and coagulation cascade (**Figure 3C**). Furthermore KEGG pathway analysis also suggested an involvement of arachidonic acid metabolism to be modulated in AMD. Literature mining also revealed a semantic link between cataract formation and up-regulated levels of GPX3 in the lens.

## Discussion

The aim of this pilot study was to test the hypothesis that specific proteins in vitreous samples of patients with exudative AMD are significantly associated with the disease and may lead to a better understanding of the pathophysiology of the disease.

We initially set out to analyze native peptides from vitreous (top-down strategy) with a mass of up to 20 kDa using CE-MS analysis. However, this strategy did not result in satisfactory data, as a result of high variability (Koss M et al. Proteomics in AMD; Poster 2927/A327 at the annual meeting of the Assiciation for Research in Vision and Ophtalmology (AVRO), Ft.Lauderdale, USA in 2012). A possible reason for the large variation between the measurements could be the presence of hyaluronic acid. Because of the highly negative charge of its monomers, hyaluronic acid reacts with positively charged proteins such as albumin to build up polyelectrolyte complexes displaying low solubility [32]. Since the top-down strategy failed to give the expected results, we changed the strategy and in the current study employed trypsin digested samples for proteome analysis of vitreous.

Using this bottom-up approach we could successfully analyze the vitreous humor samples with CE-MS, including fragments of proteins with molecular masses above 20 kDa. We identified a total of 97 proteins, 19 of those significantly increased in AMD patients. We selected 5 random proteins among these 19 for validation by Western blot analysis. One protein (alpha-1-antitrypsin) reached statistical significance while two others (transthyretin and apoliprotein A1) displayed a non-significant but increased abundance in AMD. The latter is most likely due to the low number of patients in the validation set (n = 4/group) and needs validation.

Most of the upregulated proteins in AMD patients are plasma proteins. Our findings therefore may, to some degree, be representative of the leakage from exudative CNV into the vitreous. Even though plasma proteins might not be responsible for the onset of an exudative AMD, their levels might be of diagnostic value for disease and represent the underlying pathophysiology of AMD. Future studies are important to identify which of these proteins are secreted from the abnormal CNV tissue.

The observed upregulation of a number of proteins in vitreous of AMD patients provides further insights into the pathophysiology of AMD:

### Transport proteins

Upregulation of transport proteins such as albumin and serotransferrin in AMD patients confirms the findings from previous proteome analyses of anterior chamber fluids [5]. In addition, further transport proteins like transthyretin and apolipoprotein A-I were identified. All of these were upregulated in the AMD group; all are biologically essential for cell homeostasis (albumin) and are carriers of hormones like retinol (albumin and transthyretin) or ions (serotransferrin). It is important to stress that in chronic diseases, such as AMD, the dysregulation of these abundant proteins has been observed in serum [33], [34], but so far, their presence in the vitreous has received little attention.

### Inflammatory proteins

Immunoglobulin heavy chains and alpha-1-antitrypsin were upregulated in the AMD samples. Alpha-1-antitrypsin is an acute phase protein member of the serpin family that inhibits a wide variety of proteases and thereby protects tissues from enzymes of inflammatory cells, especially neutrophil elastase. This is the first time that increased alpha-1-antitrypsin abundance in vitreous has been associated with AMD. However alpha-1-antitrypsin does not seem specific for AMD since upregulation of alpha-1-antitrypsin was observed in both aqueous and blood of patients with glaucoma [35].

### Mediators of coagulation

Fibrinogen is essential during coagulation as it is converted by plasmin into fibrin, and higher blood levels have been associated with AMD [36]. Rheopheresis, a treatment that microfilters fibrinogen from the blood of AMD patients, seems to be protective in the progression of AMD, however, the approach is controversial discussed [37]. In this study, we demonstrated that the fibrinogen alpha chain was upregulated in the AMD group. As for alpha-1-antitrypsin, although potentially involved in the pathophysiology of AMD, increased fibrinogen abundance in vitreous is not specific for AMD since such upregulation was previously also observed in vitreous samples of patients with diabetic retinopathy [38].

### Specific ocular markers

In humans, prostaglandin H2-D isomerase catalyzes the conversion of prostaglandin H2 to prostaglandin D2, which functions as a neuromodulator and as a trophic factor in the central nervous system for fatty acid biosynthesis. Its occurrence has been described in proliferative diabetic retinopathy; but it has not been previously associated with AMD [39]. The inter-photoreceptor retinol binding protein is a large glycoprotein, located in the extracellular matrix between the RPE and the photoreceptors. It is known to bind retinoids and is thought to transport retinoids between the retinal pigment epithelium and the photoreceptors. Its potential involvement in AMD has recently been described based on the analysis of blood samples [40], [41] and is confirmed by our findings.

The bioinformatic analyses using GO-term clustering, physical interaction module assembly, and KEGG pathway data mapping have shown an involvement of pathways consisting of fatty acid binding and transport, exocytosis, and protease inhibitory activity, which are also partially involved in Complement and coagulation cascades. However, more importantly is the notion that there appears to be an up-regulation of hydrogen peroxide catabolic processes, which suggest that oxidative stress is exerted in AMD.

The interactome analysis surprisingly showed a highly interconnected network of 18 of the 19 relevant proteins, where only 36 additional molecules were needed to generate a specific molecular network, suggesting that the up-regulated proteins form a potential dysregulated functional cluster encompassing immune responses and complement cascade proteins, protease cascades maybe linked to the membrane attack complex or possibly to counteract the enzymatic effect of up-regulated molecules such as GPX3, RBP3 and PTGDS, or as modulators of gene activation cascades. This unusually high selectivity also argues against the hypothesis that these proteins are observed in increased abundance merely as a result of unspecific leakage of plasma proteins, and support the hypothesis that the increase in these proteins is specifically linked to AMD.

The up-regulation of GPX3 is of particular interest, since it was shown previously that cataracts contain elevated levels of oxidants such as dehydroascorbic acid (DHA), indicative of oxidative stress, linked to a potentially elevated level of extracellular glutathione peroxidase GPX3 [42]. Mechanistically, the over-expression is suggested to result in accumulated oxidants in the glutathion/ascorbic acid homeostasis cycle, leading to DHA-polymer crystal formation in the lens as well as potentially toxic breakdown products of ascorbic acid.

In conclusion, in this study we could demonstrate that a bottom-up approach combining CE-MS (for protein selection) and LC-MS/MS (for protein identification) led to the identification of 19 candidate proteins in undiluted vitreous of AMD patients. Even with the limitations of this study (e.g., demographic matching, demand for validation in a larger cohort with a wider set of clinical parameters), the novel information gained from this study of a high number of undiluted vitreous samples provides additional insight into the pathophysiology of wet AMD.
